# Neoadjuvant PD-1 blockade: a promising nonoperative strategy for mismatch repair–deficient, locally advanced rectal cancer

**DOI:** 10.1038/s41392-022-01216-3

**Published:** 2022-10-10

**Authors:** Mi Mi, Chenyang Ye, Ying Yuan

**Affiliations:** 1 Department of Medical Oncology, Key Laboratory of Cancer Prevention and Intervention, the Second Affiliated Hospital of Zhejiang University School of Medicine, 310009 Hangzhou, Zhejiang China; 2 Cancer Center, Zhejiang University, 310058 Hangzhou, Zhejiang China

**Keywords:** Gastrointestinal cancer, Tumour immunology

In a prospective phase 2 study recently published in *The New England Journal of Medicine*, Cercek et al. investigated the efficacy of the programmed death 1 (PD-1) inhibitor dostarlimab in patients with mismatch repair–deficient (dMMR), locally advanced rectal cancer (LARC). The work aimed to present preliminary evidence for the revolutionary therapeutic transition from neoadjuvant chemotherapy/radiotherapy followed by surgery to immunotherapy followed by nonoperative management in this subgroup of patients.^1^

Patients enrolled in this study were diagnosed with dMMR, stage II or III rectal cancer. The detailed enrollment criteria included age greater than 18 years, no signs of distant metastases, an Eastern Cooperative Oncology Group (ECOG) performance status score of 0 or 1, and no previous exposure to immunotherapy, chemotherapy, or radiation for the rectal tumor. The primary endpoints reported here included the overall response to neoadjuvant dostarlimab therapy and 1-year sustained clinical complete response (cCR) after completion of dostarlimab therapy. Sixteen patients were recruited and treated with dostarlimab. Twelve of these patients have received the drug for longer than 6 months and have completed the nine planned cycles of dostarlimab. The percentage of the 12 consecutive patients achieving a cCR was 100% (95% confidence interval [CI], 74 to 100), and tumor eradication was observed using endoscopy, rectal magnetic resonance imaging, and 18F-fluorodeoxyglucose–positron-emission tomography. During the median follow-up period of one year, no patients required surgery, radiotherapy or chemotherapy. To date, 4 patients have achieved 12 months of sustained cCR after the completion of dostarlimab treatment alone. Acceptable toxicity occurred in 12 of the 16 patients (75%; 95% CI, 48 to 92) without any grade 3 or higher adverse events.

**Fig. 1:**
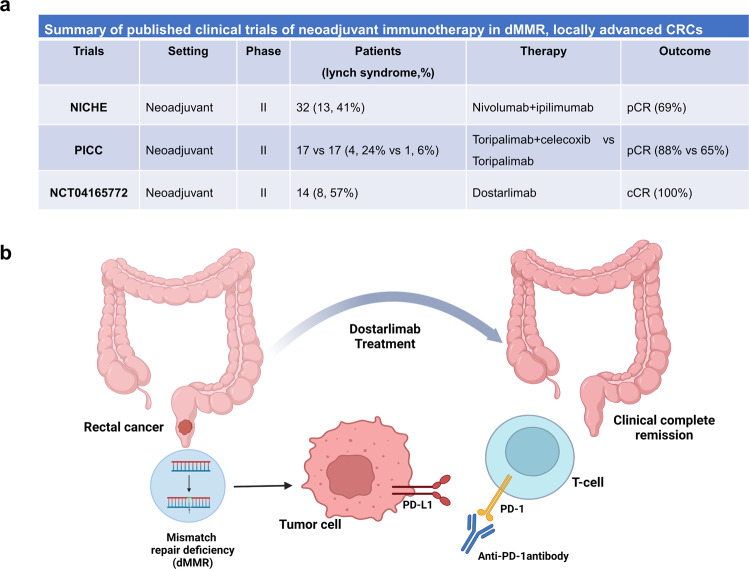
PD-1 blockade as neoadjuvant therapy in DNA dismatch repair–deficient colorectal cancers. **a** Summary of published clinical trials of neoadjuvant immunotherapy in dMMR, locally advanced CRCs. **b** The administration of anti-PD-1 antibody dostarlimab achieved clinical complete remission in the patients with dMMR, locally advanced rectal cancer. dMMR mismatch repair-deficiency, CRC colorectal cancer, pCR pathological complete response, cCR clinical complete response, PD-1 programmed death protein 1, PD-L1 programmed death ligand-1. Panel **b** was generated on Biorender.com

Patients with stage II or III rectal cancer routinely undergo neoadjuvant chemoradiotherapy followed by surgery. The quality of life of patients is permanently impacted by both surgery and chemoradiotherapy, affecting bowel and bladder function, as well as fertility. Some patients with lower rectal cancer will be surgically equipped with a permanent artificial anus for life. Since immunotherapy has been approved as a first-line treatment for metastatic dMMR colorectal cancer (CRC), researchers are debating whether immunotherapy could be used to improve the treatment modalities for dMMR, locally advanced CRCs. Several studies have explored the merits of neoadjuvant immunotherapy for dMMR, locally advanced CRCs (Fig. 1a). NICHE is the first trial of neoadjuvant immunotherapy for CRC, in which a single dose of ipilimumab combined with two doses of nivolumab were administered before surgery, with a response rate of 100% and pathological complete response (pCR) rate of 69% in 32 patients with dMMR, stage III CRC, and all patients underwent radical resections without delay (in 6 weeks).^2^ In this study, Cercek et al. reported treatment with single-agent dostarlimab (lasted for 6 months) with a 100% cCR in 12 patients with dMMR, LARC who had not undergone radiation or surgery. The remarkable results of these studies add to the literature about the potential efficacy of neoadjuvant immunotherapy. However, the difference in responses among these studies prompted us to consider establishing more appropriate subgroups that might benefit from immunotherapy.

Lynch syndrome (LS), one of the most common hereditary cancer syndromes, causes 3–5% of MSI CRCs.^3^ Controversy exists regarding whether LS CRC is more sensitive to immunotherapy. Although some studies indicated no significant difference in the immune response between patients with sporadic dMMR CRC and LS CRC in small subgroup analyses,^4,5^ other studies showed that this patient population tends to exhibit stronger immunological reactions due to the increased somatic mutational load, greater neoantigen burden and more abundant infiltrating lymphocytes in the tumor.^3^ The percentages of LS CRC in the NICHE trial and Cercek’s study were 41 and 57%, respectively. Therefore, LS CRC might represent a confounding factor, contributing to the high response rate of immunotherapy. Larger prospective studies specifically assessing the response to immunotherapy of patients with LS CRC are needed.

In addition, the response of dMMR, LARCs to PD-1 inhibitors is critically different from that of metastatic MSI-H/dMMR CRCs. The results of the KEYNOTE-177 trial showed that the percentage of patients with metastatic dMMR CRCs who had not previously received any treatment and achieved an imaging-based full response was 11.1%,^6^ which inspired us to ask why these localized dMMR rectal tumors respond much more robustly than metastatic MSI-H/dMMR CRCs. In addition, considering the complexity and heterogeneity of CRC, this study also provides a new idea for selecting immunotherapy based on the anatomic location of colorectal tumors. Although MSI-H is more common in right-side colon cancer than in left-side CRCs, a subgroup analysis based on KEYNOTE-177 was not sufficiently powerful to assess the differences between these two cancer types in terms of the efficacy of immunotherapy. In addition, researchers have not clearly determined whether the differences between rectal and colon cancer caused the discrepancy in the efficacy of immunotherapy in these two cancer subgroups. Cercek and colleagues speculated that in addition to tumor cell-intrinsic factors, such as clonality, aneuploidy, and mutation class, the gut microbiome might be a driving factor.^1,6^ The microbiota has been reported to play a crucial role in determining the host immunological response in patients with CRC. However, Cercek and colleagues have not analyzed the gut microbiome of patients in their study. If possible, additional microbiome tests should be considered for patients enrolled later.

For a number of reasons, the results from this study do not yet affect practice. First, this study includes a small sample and was conducted at a single center, which implies its limitations. Subgroup analyses of immunotherapy responses in patients with LS CRCs and sporadic dMMR CRCs are not yet available. Second, pathological complete response data are unavailable in this study, and the radiographic response does not seem to accurately predict the pathological complete response. Third, the median follow-up was short, and the primary endpoint in this study was the overall response, with no data on other clinically relevant outcomes, such as 3-year disease-free survival. Overall, little is known about the period needed to determine whether a clinical full response to dostarlimab is equivalent to a clinical cure, and immunotherapy combined with chemotherapy or radiation therapy may be required for patients with local or distant recurrence. Finally, this study only reported data for dostarlimab, and the potential clinical indications of other PD-1 and PD-L1 antibodies, such as nivolumab, pembrolizumab, and avelumab, in patients with rectal cancer presenting dMMR are not clear. Further studies are expected.

Improving the clinical outcomes of colorectal cancer has been a grand challenge. Cercek et al. provide insights into the efficacy of the single-agent dostarlimab, a PD-1 inhibitor, in patients with dMMR, locally advanced rectal cancer. Although it is a small and single-center study, its unprecedented results with a 100% clinical complete response suggest the possibility of extending beyond standard treatment for certain rectal cancers, such as lower rectal cancer, with the need for organ preservation. In addition, in the future, neoadjuvant PD-1 blockade must be evaluated in other dMMR tumors, such as localized pancreatic cancer, gastric cancer and prostate cancer.
